# Phytochemical Analysis, *α*-Glucosidase and Amylase Inhibitory, and Molecular Docking Studies on *Persicaria hydropiper* L. Leaves Essential Oils

**DOI:** 10.1155/2022/7924171

**Published:** 2022-01-19

**Authors:** Mater H. Mahnashi, Yahya S. Alqahtani, Bandar A. Alyami, Ali O. Alqarni, Muhammad Ayaz, Mehreen Ghufran, Farhat Ullah, Abdul Sadiq, Ihsan Ullah, Ikram Ul Haq, Mohammad Khalid, H. C. Ananda Murthy

**Affiliations:** ^1^Department of Pharmaceutical Chemistry, College of Pharmacy, Najran University, Najran, Saudi Arabia; ^2^Department of Pharmacy, Faculty of Biological Sciences, University of Malakand, Chakdara, 18000 Dir (L), KP, Pakistan; ^3^Department of Biochemistry, UCS, Shankar, Abdul Wali Khan University, Mardan 23200, Pakistan; ^4^Department of Pharmacy, University of Swabi, Swabi, Pakistan; ^5^National Institute of Health, Islamabad, Pakistan; ^6^Department of Pharmacognosy, College of Pharmacy, Prince Sattam Bin Abdulaziz University, Al-Kharj 11942, Saudi Arabia; ^7^Department of Applied Chemistry, School of Applied Natural Science, Adama Science and Technology University, P O Box 1888, Adama, Ethiopia

## Abstract

**Objective:**

Medicinal plants and essentials oils are well known for diverse biological activities including antidiabetic potential. This study was designed to isolate essential oils from the leaves of *Persicaria hydropiper* L. (*P. hydropiper*), perform its phytochemical analysis, and explore its in vitro antidiabetic effects.

**Materials and Methods:**

*P. hydropiper* leaves essential oils (Ph.Los) were extracted using a hydrodistillation apparatus and were subjected to phytochemical analysis using the gas chromatography mass spectrometry (GC-MS) technique. Ph.Lo was tested against two vital enzymes including *α*-glucosidase and *α*-amylase which are important targets in type-2 diabetes. The identified compounds were tested using *in silico* approaches for their binding affinities against the enzyme targets using MOE-Dock software.

**Results:**

GC-MS analysis revealed the presence of 141 compounds among which dihydro-alpha-ionone, cis-geranylacetone, *α*-bulnesene, nerolidol, *β*-caryophyllene epoxide, and decahydronaphthalene were the most abundant compounds. Ph.Lo exhibited considerable inhibitory potential against *α*-glucosidase enzyme with 70% inhibition at 1000 *μ*g mL^−1^ which was the highest tested concentration. The inhibitory activity of positive control acarbose was 77.30 ± 0.61% at the same tested concentration. Ph.Lo and acarbose exhibited IC_50_ of 170 and 18 µg mL^−1^ correspondingly. Furthermore, dose-dependent inhibitions were observed for Ph.Lo against *α*-amylase enzyme with an IC_50_ of 890 *μ*g mL^−1^. The top-ranked docking conformation was observed for *β*-caryophyllene epoxide with a docking score of -8.3182 against *α*-glucosidase, and it has established seven hydrogen bonds and one H-pi interaction at the active site residues (Phe 177, Glu 276, Arg 312, Asp 349, Gln 350, Asp 408, and Arg 439). Majority of the identified compounds fit well in the binding pocket of Tyr 62, Asp 197, Glu 233, Asp 300, His 305, and Ala 307 active residues of *α*-amylase. *β*-Caryophyllene epoxide was found to be the most active inhibitor with a docking score of -8.3050 and formed five hydrogen bonds at the active site residues of *α*-amylase. Asp 197, Glu 233, and Asp 300 active residues were observed to be making polar interactions with the ligand.

**Conclusions:**

The current study revealed that Ph.Lo is rich in bioactive metabolites which might contribute to its enzyme inhibitory potential. Inhibition of these enzymes is the key target in reducing postprandial hyperglycemia. However, further detailed in vivo studies are required for their biological and therapeutic activities.

## 1. Introduction

Diabetes mellitus (DM) is a metabolic syndrome associated with hyperglycemia due to the body's inability to produce sufficient amount of insulin or abnormalities in its secretion or tissue resistance to its action [1, 2]. Hyperglycemia in DM may also occur due to defects in the metabolic processes involved in processing carbohydrates, proteins, and fats [3, 4]. This results in development of some classical symptoms including polyuria, polydipsia, and polyphagia [5]. These metabolic abnormalities are due to low insulin level or resistance of target tissues (adipose tissue, skeletal muscles, and liver) to insulin at the level of signal transduction, insulin receptors, genes, or effecter enzymes [6]. In DM, elevated level of blood glucose for a long time is associated with a number of acute or chronic complications [7]. Globally, it has been estimated that the occurrence of diabetes has increased from 4% in 1995 to 5.4% by the year 2025 [8]. The overall prevalence as reported by the International Diabetes Federation (IDF) in 2011 was increased to 366 million people and is supposed to increase up to 552 million people by the year 2030 [9]. Furthermore, it has also been reported that 450 million people have been suffering from DM globally and the prevalence is expected to rise to 690 million by the year 2044 [10].

Regarding type-2 diabetes, targeting enzymes involved in processing dietary carbohydrates in the intestinal tract is among the vital targets. Among these, *α*-amylase and *α*-glucosidase enzymes are of high pharmacological interest and are used to control elevated glucose level in T2DM. These enzymes cause metabolic breakdown of complex dietary carbohydrates to simple sugars which are subsequently absorbed [11]. Long-chain carbohydrates are broken down into glucose by alpha-amylase enzyme, whereas *α*-glucosidase is responsible for the breakdown of disaccharides and starch into simpler monosaccharide glucose, resulting in hyperglycemia [12].

The use of medicinal plants and natural products is still a major source of therapy in the developing countries [13–15]. The discovery of modern analytical techniques has further eased the process of ethnomedicinal drug discovery to identify, isolate, and characterize target molecules [16–18]. Approximately more than four hundred plants are identified having antidiabetic potential, but only few of these plants have received medical and scientific evaluation [19]. A large number of *α*-amylase and *α*-glucosidase inhibitors are produced by different microorganisms and plants to regulate the activities of these enzymes [20]. The natural *α*-glucosidase inhibitors from plant sources, whose *α*-glucosidase inhibitory activities have been reported previously, include alkaloids, flavonoids, anthocyanins, terpenoids, curcuminoids, and phenolic compounds [21]. Miglitol, voglibose, and acarbose are the only three *α*-glucosidase inhibitors which are in clinical practice presently for the treatment of patients with T2DM [22].

*Persicaria hydropiper* L. belongs to the family Polygonaceae (smartweed family) which consists of about fifty genera and twelve hundred species. It is ethnopharmacologically famous for its use as a diuretic, anti-inflammatory agent, stomachic, central nervous system (CNS) stimulant, and natural remedy in other gastrointestinal disorders [23]. *P. hydropiper* contains flavonoids, chalcone derivatives, phenylpropanoid derivatives, phenolic compounds, anthraquinone, isocoumarin, terpenoids, and steroids [24]. Previously, crude extracts and isolated compounds were reported for neuroprotective [25, 26], cytotoxic [27, 28], antimicrobial [29], gastroprotective [30], and toxicological potential [23, 31]. The current study aimed to isolate essential oils from the leaves of *P. hydropiper* and evaluate its detailed composition via gas chromatography mass spectrometry (GC-MS). Also, the study analyses the essential oils against two important targets of the type-2 diabetes, *α*-glucosidase and *α*-amylase and dock the identified compounds against these enzymes.

## 2. Materials and Methods

### 2.1. Plant Collection and Extraction of Essential Oils from Leaves

Fresh leaves from the plant were collected in 2014 from the village of Talash (Dir), KP Pakistan, and authenticated via a botanical taxonomist and curator at the botanical garden in the University of Malakand. For preservation, dried compressed leaves were submitted to the herbarium with reference no H.UOM.BG.107. Fresh leaves were then carefully rinsed using distilled water and were processed via a Clevenger apparatus to isolate essential oils [32]. In brief, leaves were macerated followed by hydrodistillation in a Clevenger apparatus coupled with a condenser. Hydrodistillation was continued for three days at 100°C until a sufficient amount of essential oil was collected. Yellowish oil was collected in air-tight glass bottles and was refrigerated before being used for analysis and other assays.

### 2.2. GC-MS Analysis

GC-MS analysis of essential oils was performed via an Agilent USB-393752 gas chromatograph (Agilent Technologies, Palo Alto, CA, USA) having a HHP-5MS 5% phenylmethylsiloxane capillary column (Restek, Bellefonte, PA) with 30 m × 0.25 mm × 0.25 *μ*m film thickness and coupled with a mass spectrometer. Initially, oven temperature was sustained at 70°C for one minute, gradually increased to 180°C (at 6°C/min increase), and finally maintained at 280°C for twenty minutes. Temperatures of both the injector and detector were set at 220°C and 290°C, respectively. Helium was used as the carrier gas with a flow rate of 1 ml/min, and diluted Ph.Lo samples (1/1000 in *n*-pentane, v/v) were injected in the split-less mode. Components of the Ph.Lo were identified via comparison of their retention time (RT) with already reported spectral data in NIST, NIH, and Wiley libraries [33]. Moreover, comparison of the fragmentation pattern of mass spectra was done with the published literature [34].

### 2.3. *α*-Glucosidase Inhibitory Studies

Enzyme inhibitory potential of Ph.Lo was obtained according to the previously reported standard protocol [35]. Baker's yeast alpha-glucosidase, substrate (P-nitrophenyl-*α*-D-glucopyranoside), and control (acarbose) were acquired from authentic sources of Sigma Aldrich (USA). Enzyme solution (100 mM) was prepared using phosphate buffer of pH 6.8. Ph.Lo solutions were prepared using a small amount of surfactants (31.25–1000 *μ*g mL^−1^) in 320 *μ*l of 100 mM phosphate buffer and were kept for five minutes at 30°C. Subsequently, 3 ml (50 mM) of NaOH solution was mixed with it, and using a spectrophotometer, absorbency rates were recorded at 410 nm. Control solution consisted of all ingredients except the inhibitor (sample). Positive control was acarbose. Percent enzyme inhibitions were derived from the data using the given formula.

(1) $$\begin{array}{c} \text{“}\%\text{ Inhibition”}=\left[\frac{\text{Absorbance of Control}-\text{Absorbance of Sample}}{\text{Absorbance of control}}\right]\times 100. \end{array}$$

### 2.4. *α*-Amylase Inhibitory Studies

Likewise, *α*-amylase inhibitory studies were performed following the already established procedure [12]. In brief, 20 *μ*l enzyme was mixed in 200 *μ*l of 0.02 M sodium phosphate buffer mixed with the plant extracts (test compounds) of varying concentration ranges of 31.25–1000 *μ*g m L^−1^. The assay mixtures were then maintained at 25 ± 3°C for about ten minutes, and 200 *μ*l of starch was added to it. To terminate the reaction, 400 *μ*l of DNS reagent (dinitrosalicylic acid) was transferred to the mixture. The resultant solution was kept in a boiling water bath for five minutes and cooled. After cooling, 15 ml of distilled water was added to dilute the mixture and the absorbance was noted at 540 nm. Standard drug was acarbose, and enzyme inhibition was determined via the formula.

### 2.5. Molecular Docking Studies

The identified compounds were docked for their binding capacity in the enzymes protein pocket via MOE-Dock tool in molecular operating environment (MOE) (http://www.chemcomp.com) [36, 37]. Due to unavailability of *α*-glucosidase crystal structure, a previously reported homology model was used [38], whereas the *α*-amylase (4W93) 3D crystal structure was obtained from the Protein Databank (PDB). Before starting the docking process, the water molecules and ions present in crystal structures were removed via MOE. Thereafter, protein structures were added to hydrogen atoms via 3D protonation with subsequent minimization of energy via MOE default parameters including the gradient of 0.05 and Force Field Amber99.

Target compound structures were generated in MOE, and using the software default parameters, the energy was minimized. The selected enzymes including *α*-glucosidase and *α*-amylase subjected to docking with the identified compounds via the MOE parameters including Placement: Triangle Matcher, Rescoring: London dG. At least 10 confirmations were generated for every ligand. Subsequently, for each compound, top-ranked confirmations were developed and were subjected to further analysis. Finally, those docking results having comparatively good poses with polar, arene-arene, H-pi, and pi-H interactions were analyzed via Pymol software.

### 2.6. Statistical Analysis

Statistical analyses were performed using one-way analysis of variance (ANOVA) followed by Dunnett's test. The results are presented as the means ± SEM of triplicate observations. *P* values < 0.05 were considered as statistically significant. GraphPad Prism software (version 5) (USA) was used for the data analysis and figure creation.

## 3. Results and Discussion

### 3.1. GC-MS Analysis

**Figure 1:**
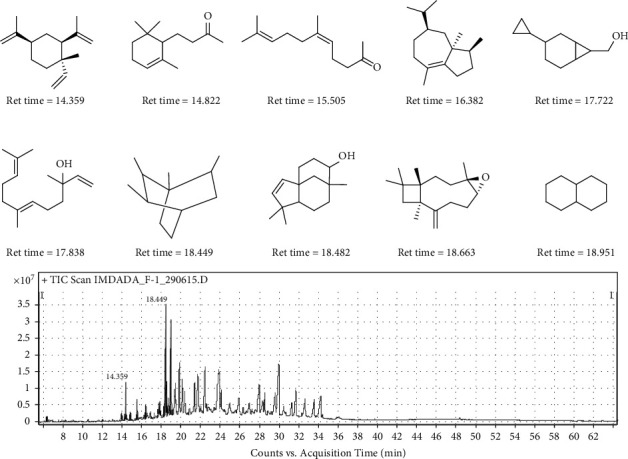
Representative image for the most abundant identified compounds.

In the GC-MS study, 141 compounds were recognized (Table S1), among which the most abundant compounds (File S1) were *β*-elemene (RT: 14.359, height%: 39.24, area%: 17.79, *m*/*z*: 81.1), dihydro-alpha-ionone (RT: 14.822, height%: 8.68, area%: 3.52, *m*/*z*: 43.1), cis-geranylacetone (RT: 15.505, height%: 21.89, area%: 9.7, *m*/*z*: 43.1), alpha-bulnesene (RT: 16.382, height%: 14.39, area%: 6.67, *m*/*z*: 93.1), bicyclo[4.1.0]heptane,-3-cyclopropyl,-7-hydroxymethyl, trans (RT: 17.722, height%: 12.08, area%: 7.4, *m*/*z*: 79.1), nerolidol (RT: 17.838, height%: 13.14, area%: 5.17, *m*/*z*: 69.1), bicyclo[2.2.2]oct-2-ene, 1,2,3,6-tetramethyl (RT: 18.449, height%: 94.65, area%: 94.88, *m*/*z*: 79.1), (1R,5S,8R,9 R)-4,4,8-trimethyltricyclo [6.3.1.0(1,5)] dodeca-2-en-9-ol (RT: 18.482, height%: 17.96, area%: 2.3, *m*/*z*: 161.1), *β*-caryophyllene epoxide (RT: 18.663, height%: 16.02, area%: 7.02, *m*/*z*: 83), and decahydronaphthalene (RT: 18.951, height%: 100, area%: 100, *m*/*z*:109.1) (Figure 1).

### 3.2. Enzyme Inhibition Studies

#### 3.2.1. Ph.Lo Exhibited Concentration-Dependent *α*-Glucosidase Inhibition

**Figure 2:**
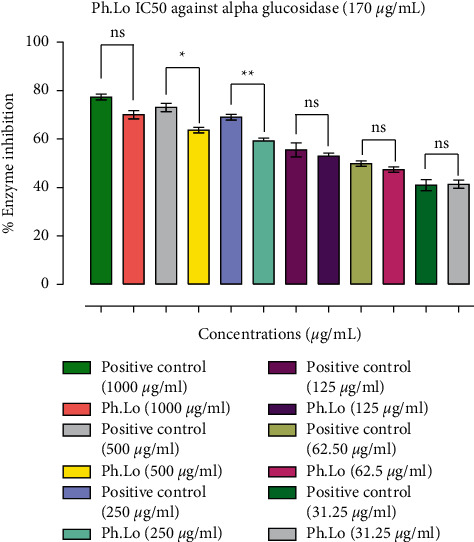
Results of *α*-glucosidase inhibition study. Data bars represent results from three independent experimental observations. Data are presented as means ± SEM. Values are significantly different (^*∗*^*p* < 0.05, ^*∗∗*^*p* < 0.01) when compared with positive control at the same tested concentrations. ns represents data groups not significantly different when compared with positive control.

In the present study, Ph.Lo was found to be highly active against *α*-glucosidase enzyme as shown in Figure 2. Ph.Lo showed inhibition rates of 70.00 ± 0.00, 63.66 ± 1.20, 59.16 ± 0.60, 53.00 ± 1.15, 47.37 ± 0.65, and 41.33 ± 1.30% at selected doses of 1000, 500, 250, 125, 62.50, and 31.25 *μ*g mL^−1^ correspondingly. The standard drug acarbose inhibitory activity showed 77.30 ± 0.61, 73.00 ± 0.00, 69.00 ± 0.00, 55.50 ± 1.04, 49.83 ± 0.44, and 41.00 ± 0.00% using the abovementioned doses, respectively. For test (Ph.Lo) and control (acarbose), IC_50_ of 170 and 18 *μ*g mL^−1^ was calculated.

#### 3.2.2. Ph.Lo Exhibits Concentration-Dependent Inhibition against *α*-Amylase Enzyme

**Figure 3:**
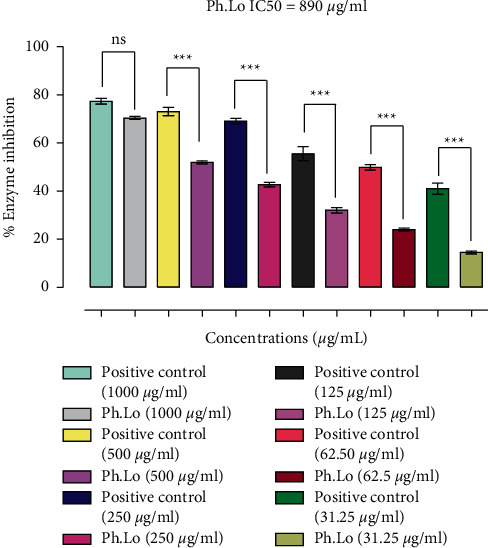
Results of *α*-amylase inhibition study. Data bars represent results from three independent experimental observations. Data are presented as means ± SEM. Values are significantly different (^*∗∗∗*^*p* < 0.001) when compared with positive control at the same tested concentrations. ns represents data groups not significantly different when compared with positive control.

Results of alpha-amylase inhibitory potential of Ph.Lo are summarized in Figure 3. Enzyme inhibitory activity of the Ph.Lo was 70.36% at 1000 µg mL^−1^, 51.91% at 500 *μ*g mL^−1^, 42.66% at 250 *μ*g mL^−1^, 32.00% at 125 *μ*g mL^−1^, 24.00% at 62.50 *μ*g mL^−1^, and 14.50% at 31.25 *μ*g mL^−1^. Positive control showed inhibition rates of 77.3% at 1000 *μ*g mL^−1^, 73.00% at 500 *μ*g mL^−1^, 69.00% at 250 *μ*g mL^−1^, 55.50% at 125 *μ*g mL^−1^, 49.83% at 62.50 *μ*g mL^−1^, and 41.00% at 31.25 *μ*g mL^−1^. Overall, concentration-dependent amylase inhibitory activities were observed for Ph.Lo as shown in Figure 3 at an IC_50_ of 890 *μ*g mL^−1^.

In GC-MS characterization, 141 phytochemicals were identified, among which dihydro-alpha-ionone, cis-geranylacetone, alpha-bulnesene, nerolidol, *β*-caryophyllene epoxide, and decahydronaphthalene were the most abundant compounds. It has been suggested by Jabeen et al. that, in a molecule, the presence of lipophilic side chain is responsible for the inhibition of alpha-glucosidase enzymes [39]. Inhibitory potential of both glucosidase and amylase enzymes has been reported previously for various volatile oils including *Eruca vesicaria* subsp. *longirostris*. Here, erucin was suggested to inhibit alpha-glucosidase. Apart from erucin, it was also reported that *β*-elemene may inhibit alpha-glucosidase activity [40]. In essential oils, the presence of monoterpenes and sesquiterpenes may contribute to inhibition of both selected enzymes [41]. Alpha-pinene, germacrene D, drimenin, and drimane-type sesquiterpene lactone are the compounds in *Hertia cheirifolia* essential oils obtained from its leaves and flowers and were suggested to contribute *α*-amylase inhibitory activity [42]. Recently, it has also been reported by Majouli et al. that *H. cheirifolia* volatile oils possess inhibitory potential against *α*-glucosidase enzyme [43]. Apart from this, inhibitory activities against both selected enzymes were reported for *Nepeta curviflora* volatile oils [44]. In these essential oils, the major phytochemical constituents include caryophyllene oxide, 1,6-dimethyl spiro-decane, and *β*-caryophyllene which are suggested for their antiamylase and antiglucosidase activities. These compounds in addition to other bioactive metabolites were identified in Ph.Lo analysis and might contribute to the overall enzyme inhibitory potential.

#### 3.2.3. Molecular Docking Studies against *α*-Amylase Enzyme

**Figure 4:**
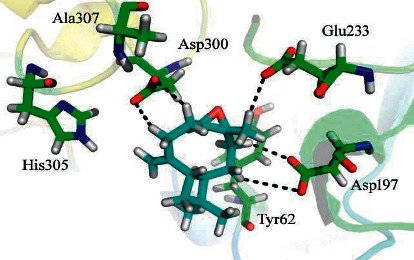
Docking conformation of *β*-caryophyllene epoxide with *α*-amylase.

Binding of the selected compounds in the binding pocket was observed (Tyr 62, Asp 197, Glu 233, Asp 300, His 305, and Ala 307 active residues) for the *α*-amylase enzyme. Docking studies revealed that the *β*-caryophyllene epoxide is the most active inhibitor with a docking score of -8.3050 and formed five hydrogen bonds with the active site residues of *α*-amylase. Asp 197, Glu 233, and Asp 300 active residues were observed to be making polar interactions with the ligand (Figure 4).

Enzyme inhibition properties of the phytochemicals might be attributed to electron-donating group (-CH3) on the identified compound. The oxygen atom of the ligand might be implicated in the considerable *in silico* performance of the compound. Interaction reports of the remaining inhibitors are given in Table 1.

#### 3.2.4. Docking with *α*-Glucosidase Enzyme

**Figure 5:**
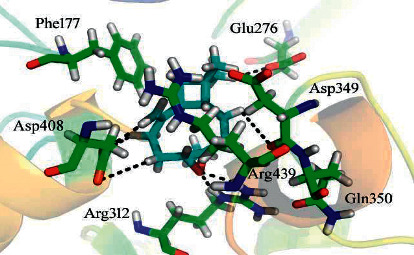
Docking conformation of *β*-caryophyllene epoxide in the active site of *α*-glucosidase.

Our simulation studies revealed that the selected phytochemicals preferentially bind with the *α*-glucosidase receptor active sites. Considerable docking conformations were observed for ß-caryophyllene epoxide with a docking score of −8.3182 which indicates that the compound established seven hydrogen bonds and one H-pi interaction with the residues of active sites (Glu 276, Phe 177, Arg 312, Asp349, Arg 439, Gln 350, and Asp 408) (Figure 5).

A considerably high inhibitory potential of the identified metabolite might be attributed to the existence of the two methyl moieties and oxygen atom attached to the (S)-2-methyloxirane moiety of the ligand (Table 2).

## 4. Conclusions

In summary, findings of this study showed that Ph.Lo is rich in bioactive phytochemicals which might contribute to the antidiabetic and health-promoting potentials of the oils. The test samples exhibited concentration-dependent inhibition of the vital enzymes implicated in the gastrointestinal absorption of postprandial glucose and thus might help in reducing the hyperglycemia in type-2 diabetes. The binding mode and energies of the identified phytochemicals against the target enzymes using the molecular docking approach further supported our claim regarding the antidiabetic potential of our test samples. Nevertheless, we suggest that, in future, in vivo studies be performed for the therapeutic and beneficial effects of these compounds in metabolism-associated disorders.

## Figures and Tables

**Table 1:** Results of the docking studies against *α*-amylase.

Compounds	Ligand		Receptor				Interaction	Distance	E (kcal/mol)	Docking scores
4-Thujanol	O	28	O	TYR	62	(A)	Hydrogen-donor	2.98	−1.3	−7.1947
	O	28	NE2	HIS	101	(A)	Hydrogen-acceptor	3.01	−1.5	
Alpha-bulnesene	C	3	OD1	ASP	197	(A)	Hydrogen-donor	3.93	−0.1	−7.3019
	C	14	OE1	GLU	233	(A)	Hydrogen-donor	3.63	−0.1	
	C	33	OD1	ASP	300	(A)	Hydrogen-donor	3.79	−0.1	
Alpha-muurolene	C	32	5-ring	HIS	101	(A)	Hydrogen-pi	4.77	−0.4	−6.7334
	C	36	5-ring	HIS	299	(A)	Hydrogen-pi	4.59	−0.3	
Beta-elemene	C	24	OD2	ASP	300	(A)	Hydrogen-donor	3.91	−0.1	−6.0798
	C	24	5-ring	HIS	305	(A)	Hydrogen-pi	4.74	−0.1	
Beta-ocimene	C	11	O	TYR	62	(A)	Hydrogen-donor	3.75	−0.1	−6.5892
	C	11	5-ring	HIS	101	(A)	Hydrogen-pi	4.03	−0.1	
	C	19	5-ring	TRP	59	(A)	Hydrogen-pi	4.66	−0.3	
Bornyl acetate	C	6	O	TYR	62	(A)	Hydrogen-donor	3.61	−0.1	−8.0205
	C	6	OD2	ASP	197	(A)	Hydrogen-donor	3.3	−0.1	
	C	25	OD1	ASP	300	(A)	Hydrogen-donor	3.75	−0.1	
	C	25	OD2	ASP	300	(A)	Hydrogen-donor	4.12	−0.1	
	O	35	NE2	HIS	299	(A)	Hydrogen-acceptor	2.96	−1.1	
	C	1	5-ring	HIS	101	(A)	Hydrogen-pi	4.52	−0.2	
	C	16	6-ring	TYR	62	(A)	Hydrogen-pi	4.75	−0.1	
Campherenone	C	1	OD1	ASP	197	(A)	Hydrogen-donor	3.86	−0.1	−5.9272
	O	23	CZ3	TRP	58	(A)	Hydrogen-acceptor	3.69	−0.1	
	O	23	NE2	HIS	299	(A)	Hydrogen-acceptor	3.3	−1.9	
	C	16	6-ring	TRP	58	(A)	Hydrogen-pi	4.87	−0.2	
Caprylic acid	O	1	OD1	ASP	197	(A)	Hydrogen-donor	3.04	−4.5	−7.5270
	O	1	OD2	ASP	197	(A)	Hydrogen-donor	2.95	−1.1	
	C	16	OD2	ASP	300	(A)	Hydrogen-donor	3.51	−0.1	
	C	19	OD1	ASP	300	(A)	Hydrogen-donor	3.85	−0.1	
Fenchol	C	21	OD1	ASP	197	(A)	Hydrogen-donor	3.71	−0.1	−6.4744
	C	25	OD2	ASP	300	(A)	Hydrogen-donor	3.7	−0.1	
	O	29	CZ3	TRP	58	(A)	Hydrogen-acceptor	3.42	−0.1	
Fixol	C	23	OD1	ASP	197	(A)	Hydrogen-donor	3.66	−0.1	−6.3792
	O	1	NE2	GLN	63	(A)	Hydrogen-acceptor	3.08	−0.4	
	O	1	5-ring	TRP	59	(A)	Hydrogen-pi	3.62	−0.1	
Isocaryophyllene	C	13	OD1	ASP	300	(A)	Hydrogen-donor	3.74	−0.1	−7.2755
	C	13	OD2	ASP	300	(A)	Hydrogen-donor	3.48	−0.1	
	C	18	OD2	ASP	300	(A)	Hydrogen-donor	3.5	−0.1	
	C	29	OE1	GLU	233	(A)	Hydrogen-donor	3.34	−0.1	
Limonene	C	5	6-ring	TYR	62	(A)	Hydrogen-pi	4.63	−0.4	−6.7494
	C	15	5-ring	HIS	299	(A)	Hydrogen-pi	4.56	−0.3	
Myrcene	C	1	OE1	GLU	233	(A)	Hydrogen-donor	3.46	−0.1	−6.1922
	C	1	OD2	ASP	300	(A)	Hydrogen-donor	3.85	−0.1	
Nerolidol	C	17	OD2	ASP	300	(A)	Hydrogen-donor	3.68	−0.1	−7.0590
	C	20	OD2	ASP	300	(A)	Hydrogen-donor	3.82	−0.1	
	C	27	OD1	ASP	197	(A)	Hydrogen-donor	3.47	−0.1	
	C	20	5-ring	HIS	299	(A)	Hydrogen-pi	4.64	−0.2	
Octylcyclopropane	C	15	OD2	ASP	197	(A)	Hydrogen-donor	4.11	−0.1	−6.9528
	C	24	OE1	GLU	233	(A)	Hydrogen-donor	3.93	−0.1	
Sativene	C	3	O	TYR	62	(A)	Hydrogen-donor	3.57	−0.1	−6.9528
	C	6	O	TYR	62	(A)	Hydrogen-donor	3.45	−0.1	
*β*-Caryophyllene epoxide	C	9	OD2	ASP	300	(A)	Hydrogen-donor	3.65	−0.1	−8.3050
	C	12	OD1	ASP	197	(A)	Hydrogen-donor	3.57	−0.1	
	C	21	OD1	ASP	197	(A)	Hydrogen-donor	3.67	−0.1	
	C	21	OE1	GLU	233	(A)	Hydrogen-donor	3.61	−0.1	
	C	38	OD1	ASP	300	(A)	Hydrogen-donor	3.88	−0.1	
Terpineol	C	9	OD1	ASP	197	(A)	Hydrogen-donor	3.76	−0.1	−7.4857
	C	12	O	TYR	62	(A)	Hydrogen-donor	3.6	−0.1	
	C	20	OE1	GLU	233	(A)	Hydrogen-donor	4.15	−0.1	
	C	24	OD1	ASP	197	(A)	Hydrogen-donor	3.58	−0.1	
	O	28	NH2	ARG	195	(A)	Hydrogen-acceptor	3	−0.4	
	O	28	NE2	HIS	299	(A)	Hydrogen-acceptor	3.17	−1.9	
	O	28	6-ring	TYR	62	(A)	Hydrogen-pi	3.87	−0.1	

**Table 2:** Results of the docking study against *α*-glucosidase.

Compounds	Ligand		Receptor			Interaction	Distance	E (kcal/mol)	Docking scores
4-Thujanol	C	1	OD2	ASP	68	Hydrogen-donor	3.26	−0.1	−8.0694
	C	1	OD2	ASP	349	Hydrogen-donor	3.76	−0.2	
	C	18	OD1	ASP	214	Hydrogen-donor	3.48	−0.1	
	O	28	OD2	ASP	68	Hydrogen-donor	2.91	−1.8	
	O	28	NH1	ARG	439	Hydrogen-acceptor	3.06	−3.4	
Alpha-bulnesene	C	1	O	ASP	349	Hydrogen-donor	3.67	−0.1	−7.6718
	C	17	O	ASP	349	Hydrogen-donor	3.57	−0.1	
	C	21	OE1	GLU	276	Hydrogen-donor	3.84	−0.1	
	C	21	OE2	GLU	276	Hydrogen-donor	3.5	−0.1	
	C	25	OD2	ASP	408	Hydrogen-donor	3.76	−0.1	
	C	3	6-ring	PHE	300	Hydrogen-pi	4.61	−0.1	
	C	30	6-ring	PHE	177	Hydrogen-pi	4.23	−0.1	
Alpha-muurolene	C	22	OD2	ASP	349	Hydrogen-donor	4.14	−0.1	−7.5763
	C	28	OE1	GLU	276	Hydrogen-donor	3.56	−0.1	
	C	28	OE2	GLU	276	Hydrogen-donor	3.62	−0.1	
	C	32	OD1	ASN	347	Hydrogen-donor	3.79	−0.1	
	C	14	6-ring	PHE	300	Hydrogen-pi	4.55	−0.1	
	C	32	6-ring	PHE	300	Hydrogen-pi	4.04	−0.3	
Beta-elemene	C	21	OE1	GLN	350	Hydrogen-donor	3.57	−0.1	−7.7074
	C	33	OE2	GLU	276	Hydrogen-donor	3.38	−0.1	
	C	36	OD2	ASP	349	Hydrogen-donor	3.81	−0.1	
	C	33	5-ring	HIS	348	Hydrogen-pi	4.26	−0.1	
Beta-ocimene	C	7	OD2	ASP	408	Hydrogen-donor	3.76	−0.1	−7.1334
	C	13	O	ASP	349	Hydrogen-donor	3.94	−0.1	
	C	19	OE1	GLN	350	Hydrogen-donor	3.86	−0.1	
	C	23	OD1	ASN	347	Hydrogen-donor	3.55	−0.1	
	C	23	O	ASP	349	Hydrogen-donor	3.41	−0.1	
	C	19	6-ring	PHE	300	Hydrogen-pi	3.53	−0.3	
	C	23	6-ring	PHE	300	Hydrogen-pi	4.55	−0.1	
Bornyl acetate	C	1	6-ring	PHE	177	Hydrogen-pi	4.06	−0.7	−7.2826
Campherenone	O	23	NH1	ARG	439	Hydrogen-acceptor	2.95	−1	−6.5621
Caprylic acid	O	1	O	ASP	349	Hydrogen-donor	2.97	−1.8	−8.0814
	C	19	OD2	ASP	349	Hydrogen-donor	3.48	−0.1	
	O	26	NE	ARG	312	Hydrogen-acceptor	3.02	−0.2	
	O	26	CE1	TYR	313	Hydrogen-acceptor	3.37	−0.1	
Fenchol	C	6	OD2	ASP	408	Hydrogen-donor	3.67	−0.1	−7.3643
	O	29	O	ASP	349	Hydrogen-donor	2.99	−1.3	
	C	21	6-ring	PHE	300	Hydrogen-pi	4.5	−0.1	
	C	25	6-ring	PHE	300	Hydrogen-pi	4.31	−0.4	
Fixol	O	1	OD2	ASP	68	Hydrogen-donor	2.94	−2.3	−7.6468
	C	4	OD1	ASP	214	Hydrogen-donor	3.87	−0.1	
	C	8	OD2	ASP	349	Hydrogen-donor	3.58	−0.1	
	C	21	OD2	ASP	408	Hydrogen-donor	3.98	−0.1	
	O	1	NH1	ARG	439	Hydrogen-acceptor	3.11	−3.2	
	C	12	6-ring	PHE	177	Hydrogen-pi	3.98	−0.2	
Isocaryophyllene	C	1	OD2	ASP	349	Hydrogen-donor	3.75	−0.1	−7.0742
	C	4	O	ASP	349	Hydrogen-donor	3.41	−0.1	
	C	25	OD2	ASP	349	Hydrogen-donor	3.83	−0.1	
	C	33	OD1	ASP	214	Hydrogen-donor	4.11	−0.1	
	C	33	OE1	GLU	276	Hydrogen-donor	3.3	−0.1	
	C	25	5-ring	HIS	348	Hydrogen-pi	4.65	−0.1	
Limonene	C	2	OE1	GLN	350	Hydrogen-donor	3.96	−0.1	−7.1971
	C	5	O	ASP	349	Hydrogen-donor	3.51	−0.1	
	C	15	O	VAL	303	Hydrogen-donor	3.82	−0.1	
	C	15	OE1	GLN	350	Hydrogen-donor	3.77	−0.1	
	C	24	OD2	ASP	408	Hydrogen-donor	3.75	−0.1	
Myrcene	C	23	O	VAL	303	Hydrogen-donor	3.35	−0.1	−7.8979
	C	23	OE1	GLN	350	Hydrogen-donor	3.4	−0.1	
Nerolidol	C	2	OE1	GLN	350	Hydrogen-donor	3.7	−0.1	−8.2988
	C	6	O	ASP	349	Hydrogen-donor	3.53	−0.1	
	C	33	OD2	ASP	68	Hydrogen-donor	3.31	−0.1	
	C	37	OD2	ASP	68	Hydrogen-donor	3.8	−0.1	
	C	37	OD2	ASP	349	Hydrogen-donor	3.59	−0.1	
	C	24	6-ring	PHE	177	Hydrogen-pi	4.78	−0.2	
	C	30	6-ring	PHE	177	Hydrogen-pi	4	−0.8	
	C	33	6-ring	PHE	177	Hydrogen-pi	4.57	−0.3	
	O	41	6-ring	PHE	300	Hydrogen-pi	3.96	−0.1	
Octylcyclopropane	C	9	OD1	ASP	214	Hydrogen-donor	3.69	−0.1	−7.5104
	C	12	OE2	GLU	276	Hydrogen-donor	4.17	−0.1	
	C	15	OD2	ASP	349	Hydrogen-donor	3.51	−0.1	
	C	21	O	ASP	349	Hydrogen-donor	3.96	−0.1	
	C	27	O	ASP	349	Hydrogen-donor	4.04	−0.1	
	C	30	OE1	GLN	350	Hydrogen-donor	3.72	−0.1	
	C	27	6-ring	PHE	300	Hydrogen-pi	3.88	−0.1	
Sativene	C	3	OE1	GLN	350	Hydrogen-donor	3.81	−0.1	−7.6121
	C	35	OD1	ASP	214	Hydrogen-donor	3.78	−0.1	
ß-Caryophyllene epoxide	C	9	OD2	ASP	408	Hydrogen-donor	3.75	−0.1	−8.3182
	C	12	O	ASP	349	Hydrogen-donor	3.52	−0.1	
	C	16	OD2	ASP	408	Hydrogen-donor	3.66	−0.1	
	C	30	OE1	GLU	276	Hydrogen-donor	3.6	−0.1	
	C	38	OD2	ASP	408	Hydrogen-donor	3.66	−0.1	
	O	25	NE	ARG	312	Hydrogen-acceptor	2.83	−3.7	
	O	25	NH2	ARG	312	Hydrogen-acceptor	2.98	−2.8	
	C	27	6-ring	PHE	177	Hydrogen-pi	3.97	−0.2	
Terpineol	C	7	OD1	ASP	214	Hydrogen-donor	3.91	−0.1	−7.8178
	C	20	OD1	ASP	214	Hydrogen-donor	3.68	−0.1	
	C	24	OE1	GLN	181	Hydrogen-donor	4.12	−0.1	
	O	28	OD2	ASP	68	Hydrogen-donor	2.93	−2.2	
	O	28	NH1	ARG	439	Hydrogen-acceptor	3.35	−1.6	
	C	4	6-ring	PHE	177	Hydrogen-pi	4.62	−0.5	
	C	24	6-ring	PHE	177	Hydrogen-pi	3.49	−0.1	

## Data Availability

The experimental data used to support the findings of this study are available from the corresponding author upon request.

## Supplementary Materials

Supplementary Materials Supplementary file S1: data related to identified compounds re provided as File S1 and Table S1 containing the list of identified compounds and their details.
