# Supplementary material for: Phytochemical Analysis, α-Glucosidase and Amylase Inhibitory, and Molecular Docking Studies on Persicaria hydropiper L. Leaves Essential Oils
Source: Evid Based Complement Alternat Med. 2022 Jan 19;2022:7924171. doi: 10.1155/2022/7924171 (PMC8791729; doi:10.1155/2022/7924171)
Supplement: Supplementary Materials — Supplementary file S1: data related to identified compounds re provided as File S1 and Table S1 containing the list of identified compounds and their details. [file 7924171.f1.docx]

**Phytochemical analysis, α-glucosidase, amylase inhibitory and molecular docking studies on *Persicaria hydropiper* L. leaves essential oils**

Mater H. Mahnashi^1^, Yahya S. Alqahtani^1^, Bandar A. Alyami^1^, Ali O. Alqarni^1^, Muhammad Ayaz*^2^, Mehreen Ghufran^3^, Farhat Ullah^2^, Abdul Sadiq^2^, Ihsan Ullah^4^, Ikram Ul Haq^5^, Mohammad Khalid^6^, HC Ananda Murthy^7^*

^1^Department of pharmaceutical chemistry, College of Pharmacy, Najran University, Najran, Kingdom of Saudi Arabia

^2^Department of Pharmacy, Faculty of Biological Sciences, University of Malakand, Chakdara, 18000 Dir (L), KP, Pakistan.

^3^Department of Biochemistry, UCS, Shankar, Abdul Wali Khan University, Mardan, Mardan-23200, Pakistan.

^4^National Institute of Health, Islamabad, Pakistan

^5^Department of Pharmacy, University of Swabi, Swabi, Pakistan

^6^Department of Pharmacognosy, College of Pharmacy, Prince Sattam Bin Abdulaziz University, Al-Kharj, 11942, Saudi Arabia

^7^Department of Applied Chemistry, School of Applied Natural Science, Adama Science and Technology University, P O Box 1888, Adama, Ethiopia.

***Correspondence;***

H C Ananda Murthy: Email: [anandkps350@gmail.com](mailto:anandkps350@gmail.com)

Muhammad Ayaz: Email: [Ayazuop@gmail.com](mailto:Ayazuop@gmail.com)

**File S1: Bioactive compounds identified in GC-MS analysis of Ph.Lo**

**Compound structure:**

**
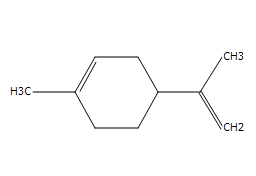
**

| **Compound Label** | **Name** | ***m/z*** | **RT** | **Algorithm** |
| --- | --- | --- | --- | --- |
| Cpd 2: dl-Limonene $$ Cyclohexene, 1-methyl-4-(1-methylethenyl)- (CAS) $$ Nesol | **dl-Limonene $$ Cyclohexene, 1-methyl-4-(1-methylethenyl)- (CAS) $$ Nesol** | 68.1 | 6.351 | Find by Chromatogram Deconvolution |

**Formula:** C10H16

**MS Spectrum:**

**Zoomed MS Spectrum:** ****

| **MS Spectrum Peak List** | | | | |
| --- | --- | --- | --- | --- |
| ***m/z*** | **Abund** |  |  |  |
| 53.1 | 81068.2 |  |  |  |
| 67.1 | 225131.3 |  |  |  |
| 68.1 | 288986.4 |  |  |  |
| 77.1 | 57796.3 |  |  |  |
| 79.1 | 102685.2 |  |  |  |
| 91.1 | 61285.1 |  |  |  |
| 92.1 | 62680.8 |  |  |  |
| 93.1 | 181474 |  |  |  |
| 94.1 | 70103.9 |  |  |  |
| 107.1 | 51526.6 |  |  |  |

**Library Spectrum:**

**Difference Spectrum:**

**Comparison Library Link:** https://webbook.nist.gov/cgi/cbook.cgi?ID=C138863&Mask=200

-------------------------------------------------------------------------------------------------------------------------------

**Compound Structure:**

**
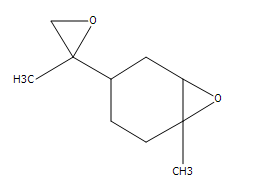
**

| **Compound Label** | **Name** | ***m/z*** | **RT** | **Algorithm** |
| --- | --- | --- | --- | --- |
| Cpd 25: LIMONENE DIOXIDE 2 | **LIMONENE DIOXIDE 2** | 43.1 | 13.77 | Find by Chromatogram Deconvolution |

**MS Spectrum:** ****

**Zoomed Mass spectrum:**

| **MS Spectrum Peak List** | | | | |
| --- | --- | --- | --- | --- |
| ***m/z*** | **Abund** |  |  |  |
| 41.2 | 29649.2 |  |  |  |
| 43.1 | 164378.9 |  |  |  |
| 53.1 | 20558 |  |  |  |
| 55.1 | 23249.5 |  |  |  |
| 67.1 | 28832.8 |  |  |  |
| 79.1 | 19417.6 |  |  |  |
| 81.1 | 32327.6 |  |  |  |
| 82.1 | 20730.4 |  |  |  |
| 93.1 | 19746.7 |  |  |  |
| 107.1 | 22312.3 |  |  |  |

**Library Spectrum:** ****

**Difference Spectrum:**

**Comparison Library Link:** https://pubchem.ncbi.nlm.nih.gov/compound/Limonene-dioxide

-------------------------------------------------------------------------------------------------------------------------------

**Compound Structure:**

**
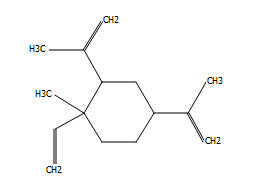
**

| **Compound Label** | **Name** | ***m/z*** | **RT** | **Algorithm** |
| --- | --- | --- | --- | --- |
| Cpd 29: (-)-.beta.-Elemene $$ CIS-1,3-DIISOPROPENYL-TRANS-4-VINYL-4-METHYLCYCLOHEXANE | **(-)-.beta.-Elemene $$ CIS-1,3-DIISOPROPENYL-TRANS-4-VINYL-4-METHYLCYCLOHEXANE** | 93.1 | 14.36 | Find by Chromatogram Deconvolution |

**MS Spectrum:** ****

**Zoomed Mass spectrum** ****

| **MS Spectrum Peak List** | | | | |
| --- | --- | --- | --- | --- |
| ***m/z*** | **Abund** |  |  |  |
| 53.1 | 433510.8 |  |  |  |
| 55.1 | 414521.2 |  |  |  |
| 67.1 | 700206.5 |  |  |  |
| 68.1 | 664511 |  |  |  |
| 79.1 | 576768.2 |  |  |  |
| 81.1 | 907553.7 |  |  |  |
| 91.1 | 425317.1 |  |  |  |
| 93.1 | 910153.9 |  |  |  |
| 107.1 | 544808.8 |  |  |  |
| 121.1 | 352549.3 |  |  |  |

**Library Spectrum:** ****

**Difference Spectrum:** ****

**Comparison Library Link:** https://pubchem.ncbi.nlm.nih.gov/compound/beta-Elemene

**---------------------------------------------------------------------------------------------------------------------------**

**Compound Structure:**

**
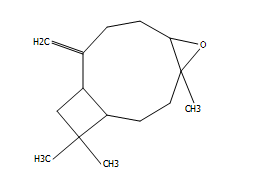
**

| **Compound Label** | **Name** | ***m/z*** | **RT** | **Algorithm** |
| --- | --- | --- | --- | --- |
| Cpd 70: (-)-Caryophyllene oxide $$ (-)-.beta.-Caryophyllene epoxide | **(-)-Caryophyllene oxide $$ (-)-.beta.-Caryophyllene epoxide** | 83.1 | 18.66 | Find by Chromatogram Deconvolution |

**MS Spectrum:** ****

**Zoomed Mass spectrum** ****

| **MS Spectrum Peak List** | | | | |
| --- | --- | --- | --- | --- |
| ***m/z*** | **Abund** |  |  |  |
| 41.2 | 225170.6 |  |  |  |
| 43.1 | 226848.2 |  |  |  |
| 55.1 | 295519.7 |  |  |  |
| 69.1 | 217183.7 |  |  |  |
| 79.1 | 224708.7 |  |  |  |
| 83.1 | 425486 |  |  |  |
| 91.1 | 230745.4 |  |  |  |
| 93.1 | 378806 |  |  |  |
| 109.1 | 268305.5 |  |  |  |
| 121.1 | 194041 |  |  |  |

**Library Spectrum:** ****

**Difference Spectrum:** ****

**Comparison Library Link:** https://webbook.nist.gov/cgi/cbook.cgi?ID=C1139306&Mask=200

------------------------------------------------------------------------------------------------------------------------

**Compound Structure:**

**
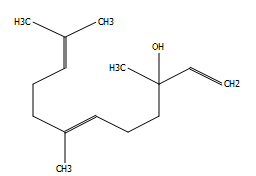
**

| **Compound Label** | **Name** | ***m/z*** | **RT** | **Algorithm** |
| --- | --- | --- | --- | --- |
| Cpd 45: Nerolidol $$ 1,6,10-Dodecatrien-3-ol, 3,7,11-trimethyl- (CAS) $$ E-farnesol | **Nerolidol $$ 1,6,10-Dodecatrien-3-ol, 3,7,11-trimethyl- (CAS) $$ E-farnesol** | 69.1 | 16.27 | Find by Chromatogram Deconvolution |

**MS Spectrum:** ****

**Zoomed Mass spectrum** ****

| **MS Spectrum Peak List** | | | | |
| --- | --- | --- | --- | --- |
| ***m/z*** | **Abund** |  |  |  |
| 41.2 | 84589.8 |  |  |  |
| 53.1 | 38743.1 |  |  |  |
| 69.1 | 154865.5 |  |  |  |
| 77.1 | 41199.5 |  |  |  |
| 79.1 | 52518.7 |  |  |  |
| 81.1 | 44691.3 |  |  |  |
| 91.1 | 42455.5 |  |  |  |
| 93.1 | 84032.4 |  |  |  |
| 123.1 | 26712.8 |  |  |  |
| 133.1 | 27806.8 |  |  |  |

**Library Spectrum:**

**Difference Spectrum:** ****

**Comparison Library Link:** https://webbook.nist.gov/cgi/cbook.cgi?ID=C142507&Mask=200

---------------------------------------------------------------------------------------------------------------------------

**Compound Structure:**

**
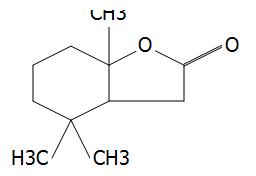
**

| **Compound Label** | **Name** | ***m/z*** | **RT** | **Algorithm** |
| --- | --- | --- | --- | --- |
| Cpd 47: 2(3H)-Benzofuranone, hexahydro-4,4,7a-trimethyl- $$ Tetrahydroactinidiolide | **2(3H)-Benzofuranone, hexahydro-4,4,7a-trimethyl- $$ Tetrahydroactinidiolide** | 43.1 | 16.54 | Find by Chromatogram Deconvolution |

**MS Spectrum:** ****

**Zoomed Mass spectrum** ****

| **MS Spectrum Peak List** | | | | |
| --- | --- | --- | --- | --- |
| ***m/z*** | **Abund** |  |  |  |
| 41.2 | 94323.7 |  |  |  |
| 43.1 | 191840.4 |  |  |  |
| 55.1 | 101566.2 |  |  |  |
| 67.1 | 94269.4 |  |  |  |
| 68.1 | 71496.4 |  |  |  |
| 69.1 | 151322.5 |  |  |  |
| 81.1 | 106414.7 |  |  |  |
| 95.1 | 85628.8 |  |  |  |
| 139.1 | 91071 |  |  |  |
| 167.1 | 64923.4 |  |  |  |

**Library Spectrum:** ****

**Difference Spectrum:** ****

**Comparison Library Link:** https://webbook.nist.gov/cgi/inchi?ID=C16778271&Mask=200

------------------------------------------------------------------------------------------------------------------------------

**Compound Structure:**

**
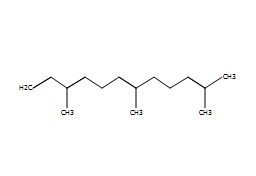
**

| **Compound Label** | **Name** | ***m/z*** | **RT** | **Algorithm** |
| --- | --- | --- | --- | --- |
| Cpd 93: .alpha.-Farnesene $$ 1,3,6,10-Dodecatetraene, 3,7,11-trimethyl- $$ Farnesene | **.alpha.-Farnesene $$ 1,3,6,10-Dodecatetraene, 3,7,11-trimethyl- $$ Farnesene** | 69.1 | 20.67 | Find by Chromatogram Deconvolution |

**MS Spectrum:**

**Zoomed Mass spectrum** ****

| **MS Spectrum Peak List** | | | | | |
| --- | --- | --- | --- | --- | --- |
| ***m/z*** | **Abund** |  |  |  |  |
| 41.2 | 96289 |  |  |  |  |
| 53.1 | 60279 |  |  |  |  |
| 55.1 | 90397 |  |  |  |  |
| 67.1 | 83051 |  |  |  |  |
| 69.1 | 140760 |  |  |  |  |
| 79.1 | 82702 |  |  |  |  |
| 91.1 | 65643 |  |  |  |  |
| 93.1 | 87672 |  |  |  |  |
| 95.1 | 85189 |  |  |  |  |
| 107.1 | 59687 |  |  |  |  |

**Library Spectrum:** ****

**Difference Spectrum:** ****

**Comparison Library Link:** https://webbook.nist.gov/cgi/cbook.cgi?ID=C502614&Mask=200

---------------------------------------------------------------------------------------------------------------------------

**Compound Structure:**

**
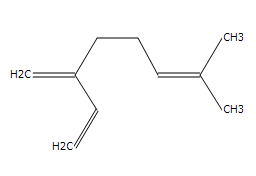
**

| **Compound Label** | **Name** | ***m/z*** | **RT** | **Algorithm** |
| --- | --- | --- | --- | --- |
| Cpd 122: myrcene $$ MYCRENE | **myrcene $$ MYCRENE** | 69.1 | 24.59 | Find by Chromatogram Deconvolution |

**MS Spectrum:** ****

**Zoomed Mass spectrum** ****

| **MS Spectrum Peak List** | | | | | |
| --- | --- | --- | --- | --- | --- |
| ***m/z*** | **Abund** |  |  |  |  |
| 41.2 | 75151 |  |  |  |  |
| 69.1 | 83759 |  |  |  |  |
| 71.1 | 39493 |  |  |  |  |
| 81.1 | 67428 |  |  |  |  |
| 93.1 | 66695 |  |  |  |  |
| 105.1 | 34878 |  |  |  |  |
| 120.1 | 11575 |  |  |  |  |
| 133.1 | 23648 |  |  |  |  |
| 136.1 | 13544 |  |  |  |  |
| 147.1 | 12749 |  |  |  |  |

**Library Spectrum:**

**Difference Spectrum:** ****

**Comparison Library Link:** https://pubchem.ncbi.nlm.nih.gov/compound/Myrcene

-------------------------------------------------------------------------------------------------------------------------------

**Compound Structure:**

**
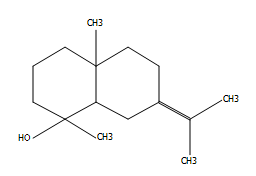
**

| **Compound Label** | **Name** | ***m/z*** | **RT** | **Algorithm** |
| --- | --- | --- | --- | --- |
| Cpd 142: junipercamphor | **junipercamphor** | 43.1 | 28.49 | Find by Chromatogram Deconvolution |

**MS Spectrum:** ****

**Zoomed Mass spectrum** ****

| **MS Spectrum Peak List** | | | | |
| --- | --- | --- | --- | --- |
| ***m/z*** | **Abund** |  |  |  |
| 43.1 | 554332.7 |  |  |  |
| 55.1 | 248931.3 |  |  |  |
| 69.1 | 266133.8 |  |  |  |
| 79.1 | 198112.2 |  |  |  |
| 81.1 | 295073.8 |  |  |  |
| 91.1 | 241227.3 |  |  |  |
| 109.1 | 281127.2 |  |  |  |
| 134.1 | 331763.9 |  |  |  |
| 189.1 | 317048.1 |  |  |  |
| 204.2 | 186986.1 |  |  |  |

**Library Spectrum:** ****

**Difference Spectrum:** ****

**Comparison Library Link:** https://pubchem.ncbi.nlm.nih.gov/compound/Juniper-camphor

**-----------------------------------------------------------------------------------------------------------------------------**

**Compound Structure:**

| **Compound Label** | **Name** | ***m/z*** | **RT** | **Algorithm** |
| --- | --- | --- | --- | --- |
| Cpd 169: Androstan-17-one, 3-ethyl-3-hydroxy-, (5.alpha.)- | **Androstan-17-one, 3-ethyl-3-hydroxy-, (5.alpha.)-** | 77.1 | 34.34 | Find by Chromatogram Deconvolution |

**MS Spectrum:** ****

**Zoomed Mass spectrum** ****

| **MS Spectrum Peak List** | | | | | |
| --- | --- | --- | --- | --- | --- |
| ***m/z*** | **Abund** |  |  |  |  |
| 55.1 | 30204 |  |  |  |  |
| 77.1 | 40699 |  |  |  |  |
| 81.1 | 33215 |  |  |  |  |
| 91.1 | 40610 |  |  |  |  |
| 93.1 | 36379 |  |  |  |  |
| 107.1 | 24830 |  |  |  |  |
| 121.1 | 24431 |  |  |  |  |
| 136.1 | 21679 |  |  |  |  |
| 149.1 | 22380 |  |  |  |  |
| 246.2 | 23126 |  |  |  |  |

**Library Spectrum:** ****

**Difference Spectrum:** ****

**Comparison Library Link:** https://webbook.nist.gov/cgi/cbook.cgi?ID=53-42-9&Units=SI

**------------------------------------------------------------------------------------------------------------------------------**

**Table S1:** Detail of compounds identified in GC-MS analysis of Ph.Lo.

| S. No. | Compound Chemical / Label Name | Common Name | RT | Formula | Hits (DB) |
| --- | --- | --- | --- | --- | --- |
| 1 | (1E)-1-(Pentyloxy)-1-butene | Not found | 6.059 | C9H18O | 10 |
| 2 | Cyclohexene, 1-methyl-4-(1-methylethenyl) | Limonene | 6.351 | C10H16 | 10 |
| 3 | 5-Oxopentanoic acid | Keto pentanoate | 6.903 | C5H8O3 | 10 |
| 4 | N-Formyl-2-hydroxy-3-methyl-2-(1-methylethyl) butanamide | Not found | 7.519 | C9H17NO3 | 10 |
| 5 | 4,4-dimethyl-1-heptene | Not found | 7.851 | C9H18 | 10 |
| 6 | 6-Methyl-2,4-heptanedione | Isovaleryl acetone | 8.222 | C8H14O2 | 10 |
| 7 | (5E)-3,6-Dimethyl-5-octen-2-one | Not found | 8.272 | C10H18O | 10 |
| 8 | 6-Methyl-5-octen-2-one | Not found | 8.819 | C9H16O | 10 |
| 9 | 1,3,3-trimethyl-2-norbornanol | Fenchol | 8.973 | C10H18O | 10 |
| 10 | trans-2,7-Dimethyl-4,6-octadien-2-ol | Not found | 9.061 | C10H18O | 10 |
| 11 | 3-Chloro-2-methyl-2-pentanol | Not found | 9.183 | C6H13ClO | 10 |
| 12 | Octanoic acid | Caprylic acid | 9.344 | C8H16O2 | 10 |
| 13 | 6-Methyl-1-heptanol | Isooctanol | 9.917 | C8H18O | 10 |
| 14 | Ethoxymethoxy cyclohexane | Not found | 10.45 | C9H18O2 | 10 |
| 15 | 2-Ethyl-1-butanol, trifluoroacetate | Not found | 10.516 | C8H13F3O2 | 10 |
| 16 | 1-(2,2-Dimethylcyclopentyl)ethanone | Not found | 10.924 | C9H16O | 10 |
| 17 | cis-13-Octadecenal | Not found | 11.116 | C18H34O | 10 |
| 18 | Octane, 1-cyclopropyl | Octylcyclopropane | 11.572 | C11H22 | 10 |
| 19 | (2S,4aS.8aR)-(-)-5,5,8a-Trimethyldecahydronaphthalene-2-yl acetate | Not found | 11.947 | C15H26O2 | 10 |
| 20 | Bornyl acetate / Borneol acetate | Endobornyl Acetate | 11.993 | C12H20O2 | 10 |
| 21 | 6-Methyl-5-octen-2-one | Not found | 12.131 | C9H16O | 10 |
| 22 | Octanal, 7-hydroxy-3,7-dimethyl | Hydroxycitronellal/ Fixol | 12.631 | C10H20O2 | 10 |
| 23 | 5,9-Tetradecadiyne | Not found | 12.999 | C14H22 | 10 |
| 24 | 2-Isopropenyl-5-methyl-6-hepten-1-ol | Not found | 13.455 | C11H20O | 10 |
| 25 | Limonene Dioxide -2 | Limonene Dioxide-2 | 13.767 | C10H16O2 | 10 |
| 26 | 2-Pentanone, 3-[(acetyloxy)methyl]-3,4-dimethyl-, (.+-.)- | Not found | 13.953 | C10H18O3 | 10 |
| 27 | Bicyclo[2.2.1]heptane-2-carboxaldehyde, 3-methyl-, (2-endo,3-exo)- | Limonene Dioxide-1 | 14.091 | C9H14O | 10 |
| 28 | Limonene Dioxide 1 | Not found | 14.201 | C10H16O2 | 10 |
| 29 | CIS-1,3-Diisopropenyl-Trans-4-Vinyl-4-Methylcyclohexane | beta.-Elemene | 14.359 | C15H24 | 10 |
| 30 | 2-Butanone, 4-(2,2-dimethyl-6-methylenecyclohexyl) | Not found | 14.446 | C13H22O | 10 |
| 31 | 4-Methylhept-6-En-3-One | Not found | 14.535 | C8H14O | 10 |
| 32 | Gamma Caryophyllene | isocaryophyllene | 14.708 | C15H24 | 10 |
| 33 | 2-Butanone, 4-(2,6,6-trimethyl-2-cyclohexen-1-yl) | Dihydro .alpha. ionone | 14.822 | C13H22O | 10 |
| 34 | Igamma Caryophyllene | isocaryophyllene | 14.988 | C15H24 | 10 |
| 35 | p-Menth-8-en-1-ol, stereoisomer | cis-.beta.-Terpineol | 15.092 | C10H18O | 10 |
| 36 | 7,8-Dihydro beta ionone | Dihydro beta ionone | 15.277 | C13H22O | 10 |
| 37 | 5,9-Undecadien-2-one, 6,10-dimethyl | cis-Geranylacetone | 15.504 | C13H22O | 10 |
| 38 | 4-Isopropenyl-1-methyl-7-oxabicyclo[4.1.0]heptan-2-one | cis-Carvone oxide | 15.648 | C10H14O2 | 10 |
| 39 | 1,3,6-Octatriene, 3,7-Dimethyl-, (E)- | Beta-Ocimene | 15.697 | C10H16 | 10 |
| 40 | (E)-3,4-Epoxy-1-(1',2'-epoxy-3',3'-epoxymethano-2',6',6'-trimethyl-1'-cycloh... | Not found | 15.751 | C15H22O3 | 10 |
| 41 | Cyclopentane, 2-ethylidene-1,1-dimethyl | Not found | 15.843 | C9H16 | 10 |
| 42 | Trans-Sabinene Hydrate | 4-Thujanol | 15.901 | C10H18O | 10 |
| 43 | (-)-Globulol | Globulol | 16.105 | C15H26O | 10 |
| 44 | 2-Heptene, 2-methyl-6-p-tolyl | Not found | 16.185 | C15H22 | 10 |
| 45 | 1,6,10-Dodecatrien-3-ol, 3,7,11-trimethyl | Nerolidol/ E-farnesol | 16.27 | C15H26O | 10 |
| 46 | Guaia-1(10),11-diene | Alpha Bulnesene | 16.382 | C15H24 | 10 |
| 47 | 2(3H)-Benzofuranone, hexahydro-4,4,7a-trimethyl | Tetrahydroactinidiolide | 16.542 | C11H18O2 | 10 |
| 48 | 1.Xi.,6.xi.,7.xi.-Cadina-4,9-diene | Alpha Muurolene | 16.591 | C15H24 | 10 |
| 49 | Alpha-Bisabolene | Bisabolene | 16.707 | C15H24 | 10 |
| 50 | 4-Isopropenyl-1-methyl-7-oxabicyclo[4.1.0]heptane | cis Limonene oxide | 16.768 | C10H16O | 10 |
| 51 | (+)-Sativen | Sativene | 16.84 | C15H24 | 10 |
| 52 | 4-Isopropenyl-1-methylcyclohexanol | Terpineol | 16.958 | C10H18O | 10 |
| 54 | Farnesyl Acetone C | Not found | 17.017 | C18H30O | 3 |
| 55 | 2(1H)-Naphthalenone, octahydro-4a,7,7-trimethyl-, ciss | Not found | 17.205 | C13H22O | 10 |
| 56 | Allyl(chloromethyl) dimethylsilane | Not found | 17.363 | C6H13ClSi | 4 |
| 57 | 3-Allyl-2,6,6-trimethylbicyclo[3.1.1]heptane | Not found | 17.413 | C13H22 | 10 |
| 58 | 1,13-Tetradecadiene | Not found | 17.594 | C14H26 | 10 |
| 59 | Bicyclo[4.1.0]heptane,-3-cyclopropyl,-7-hydroxymethyl, trans | Not found | 17.72 | C11H18O | 10 |
| 60 | cis-5-Methyl-2,3,3a,4,7,7a-hexahydroinden-1-one | Not found | 17.757 | C10H14O | 8 |
| 61 | 1,6,10-Dodecatrien-3-ol, 3,7,11-trimethyl-, [S-(Z)]-(+)-Nerolidol | Not found | 17.838 | C15H26O | 10 |
| 62 | 2H-cyclopropa[g]benzofuran, 4,5,5a,6,6a,6b-hexahydro-4,4,6b-Trimethyl | Benzofuran | 17.926 | C15H22O | 7 |
| 63 | Cyclopropanemethanol, 2-methyl-2-(4-methyl-3-pentenyl) | Not found | 18.07 | C11H20O | 10 |
| 64 | Cyclohexanol, 2-Methylene-5-(1-Methylethenyl)-, (1s-Trans) | Not found | 18.205 | C10H16O | 10 |
| 65 | Bicyclo[2.2.2]oct-2-ene, 1,2,3,6-tetramethyl | Not found | 18.448 | C12H20 | 10 |
| 66 | (1R,5S,8R,9R)-4,4,8-trimethyltricyclo[6.3.1.0(1,5)]dodeca-2-en-9-old | Not found | 18.481 | C15H24O | 10 |
| 67 | Limonene Dioxide 2 | Limonene Dioxide 2 | 18.539 | C10H16O2 | 10 |
| 68 | 3-(1-Methylhept-1-enyl)-5-methyl-2,5-dihydrofuran-2-one | Not found | 18.594 | C13H20O2 | 10 |
| 69 | (-)-.beta.-Caryophyllene epoxide | Caryophyllene oxide | 18.662 | C15H24O | 10 |
| 70 | 1,1,4,7-Tetramethyldecahydro-1H-cyclopropa[e]azulen-4-ol | Epiglobulol | 18.747 | C15H26O | 10 |
| 71 | 1-Dodecyn-3-ol | Not found | 18.931 | C12H22O | 1 |
| 72 | Naphthalene, decahydro | Decahydronaphthalene | 18.951 | C10H18 | 10 |
| 73 | 7,8-Epoxy-.alpha.-ionone | Not found | 19.056 | C13H20O2 | 10 |
| 74 | 3-Heptadecen-5-yne, (Z)- | Not found | 19.162 | C17H30 | 10 |
| 75 | Geranyl acetate [trans-3,7-dimethyl-2,6-octadien-1-ylacetate] | Not found | 19.271 | C12H20O2 | 3 |
| 76 | cis-3-Hexenyl phenyl acetate | Benzeneacetic acid | 19.316 | C14H18O2 | 10 |
| 77 | (-)-Caryophyllene oxide | Caryophyllene epoxide | 19.393 | C15H24O | 10 |
| 78 | 7,8,8a,9-Tetrahydro-8a-methylnaphtho[2,3-b]furan-4,5(4aH,6H)-dione | Not found | 19.427 | C13H14O3 | 10 |
| 79 | Veridiflorol | Veridiflorol | 19.519 | C15H26O | 10 |
| 80 | cyclohexane, 1,5-diethenyl-2,3-dimethyl-, (1.alpha.,2.beta.,3.beta.,5.beta.)- | Not found | 19.784 | C12H20 | 10 |
| 81 | 1-(3,3-dimethyl-bicyclo[2.2.1.]hept-2-yl)-2-penten-1-one | Not found | 19.973 | C14H22O | 1 |
| 82 | 2,6,10,14-Tetramethyl-2R,3R-epoxy-6E,10E,14E-hexadecatrien-1,16-diol | Not found | 20.095 | C20H34O3 | 10 |
| 83 | 8(12)-Drimen-7.alpha.,11-diol | Not found | 20.126 | C15H26O2 | 10 |
| 84 | 6-exo-Methoxy-3-methyl-3a,6a-dihydrofuro[3,4-d]isoxazol-4(6H)-one | Not found | 20.215 | C7H9NO4 | 1 |
| 85 | 11-Hexadecyn-1-ol | Not found | 20.338 | C16H30O | 10 |
| 86 | 2,6-Dimethyl-4-oxo-11-oxatricyclo[5.4.2.1(7,10).0(5,10)]undec-8-ene | Not found | 20.381 | C12H16O2 | 5 |
| 87 | 1,3,6,10-Dodecatetraene, 3,7,11-trimethyl- | Farnesene | 20.669 | C15H24 | 10 |
| 88 | Bergamotol, Z-.alpha.-trans- | Bergamotol | 20.826 | C15H24O | 10 |
| 89 | Pyrimidine, 4-cyclopropyl- | Cyclopropyl pyrimidine | 20.909 | C7H8N2 | 6 |
| 90: | 7-Oxabicyclo[4.1.0]heptane, 1-methyl-4-(2-methyloxiranyl)- | Dipentene dioxide | 21.338 | C10H16O2 | 10 |
| 91: | 5-Methylenedec-9-en-2-one | Not found | 21.645 | C11H18O | 10 |
| 92: | Bi-1-cycloocten-1-yl | Not found | 21.883 | C16H26 | 10 |
| 93: | Limonene Dioxide 2 | Limonene Dioxide 2 | 21.966 | C10H16O2 | 10 |
| 94: | Limonene Dioxide 4 | Limonene Dioxide 4 | 22.123 | C10H16O2 | 10 |
| 95: | Benzene, 4-fluoro-1,2-dimethyl- | 4-fluoro-1,2-xylene | 22.383 | C8H9F | 10 |
| 96: | 1-Pentyn-3-one, 4-methyl- | 2-Methyl-3-Oxo-4-Pentyne | 22.419 | C6H8O | 1 |
| 97 | Methyl 10,12-heptadecadiynoate | Not found | 22.507 | C18H28O2 | 10 |
| 98 | 2,6,10,14,18,22-Tetracosahexaene, 2,6,10,15,19,23-hexamethyl-, (all-E)- | Not found | 22.567 | C30H50 | 2 |
| 99 | 5-Hexyl-3,3-dimethyl-1-cyclopentene | Not found | 22.754 | C13H24 | 10 |
| 100 | (7E)-4,8,12-Trimethyltrideca-2,3,7,11,-tetraene-1-ol | NF | 22.917 | C16H26O | 3 |
| 101 | (-)-Campherenone | Campherenone | 23.016 | C15H24O | 5 |
| 102 | 5,8-methano-3,4,4a,5,6,7,8,8a-octahydro-1H-2-benzopyran-1-one | Not found | 23.104 | C10H14O2 | 1 |
| 103 | 1-(hydroxymethyl)-2-vinylcyclopentane | Not found | 23.21 | C8H14O | 10 |
| 104 | 3,4-Dimethylhexanedial | Not found | 23.431 | C8H10O2 | 10 |
| 105 | 4-Isopropenyl-4,7-dimethyl-1-oxaspiro[2.5]octane | Not found | 24.037 | C12H20O | 10 |
| 106 | 6-Methyl-2-heptanone | Not found | 24.358 | C8H16O | 10 |
| 107 | Bicyclo[4.3.0]nonane, 7-methylene-2,4,4-trimethyl-2-vinyl- | Not found | 24.449 | C15H24 | 10 |
| 108 | myrcene | Mycrene | 24.592 | C10H16 | 1 |
| 109 | 5-Isopropyl-3-phenyl-2-thiohydantoin | Not found | 24.776 | C12H14N2OS | 1 |
| 110 | 4-[Acetylmethyl]-3,3-dimethyl-3,4-dihydrobenzopyran-2H-2-one | Not found | 24.91 | C14H16O3 | 1 |
| 111 | 15-Chloro-4-pentadecyne | Not found | 25.483 | C15H27Cl | 10 |
| 112 | longipinanol | longipinanol | 25.663 | C15H26O | 3 |
| 113 | 1-Methyl-6-(3-methylbuta-1,3-dienyl)-7-oxabicyclo[4.1.0]heptane | Not found | 25.831 | C12H18O | 10 |
| 114 | 3-Buten-2-ol, 3-methyl-4-(2,6,6-trimethyl-2-cyclohexen-1-yl)- | Not found | 26.258 | C14H24O | 10 |
| 115 | 7,7-dichlorobicyclo[3.2.0]hept-2-en-6-one | Not found | 26.634 | C15H24O | 10 |
| 116 | 2-Isopropyl-tricyclo[4.3.1.1(2,5)]undec-3-en-10-ol | Not found | 26.819 | C14H22O | 10 |
| 117 | But-3-enal, 2-methyl-4-(2,6,6-trimethyl-1-cyclohexenyl)- | Not found | 27.177 | C14H22O | 10 |
| 118 | 6,6,10-Trimethylundeca-3,8,10-triene-2,7-dione | Not found | 27.3 | C14H20O2 | 3 |
| 119 | 5-Iminopyrrolidine-2-carbonitrile | Not found | 27.746 | C5H7N3 | 7 |
| 120 | 7,7-dichlorobicyclo[3.2.0]hept-2-en-6-one | Not found | 27.908 | C15H24O | 10 |
| 121 | 1,3,5-Cycloheptatriene, 3,4-diethyl-7,7-dimethyl- | Not found | 28.274 | C13H20 | 10 |
| 122 | juniper camphor | Juniper camphor | 28.487 | C15H26O | 10 |
| 123 | Longifolenaldehyde | longifolenaldehyde | 28.782 | C15H24O | 10 |
| 124 | (E)-3-(3'-methyl-1',3'-butadienyl)-2,4,4-trimethylcyclohexanone | Not found | 29.125 | C14H22O | 10 |
| 125 | N-2-Methylpropanoyl-3,4-dihydro-1H-2,1-benzoxazine | Not found | 29.524 | C12H15NO2 | 2 |
| 126 | Benzo[e]isobenzofuran-1,4-dione,1,3,4,5,5a,6,7,8,9,9a- decahydro-6,6,9a-trime | Not found | 29.9 | C15H20O3 | 10 |
| 127 | (+-)cis-1,2-Dihydroxy-1,2,3,4-tetrahydrochrysene | Not found | 29.922 | C18H16O2 | 5 |
| 128 | 3h-Cyclodeca[B]Furan-2-One, 4,9-Dihydroxy-6-Methyl-3,10-Dimethylene-3a | Not found | 30.391 | C15H20O4 | 10 |
| 129 | Cyclopentanecarboxaldehyde, 2-methyl-3-methylene- | Not found | 30.596 | C8H12O | 10 |
| 130 | Cycloisolongifolene | Cycloisolongifolene | 31.629 | C15H24 | 10 |
| 131 | aromadendrene 2 | aromadendrene | 31.933 | C15H24 | 10 |
| 132: | Cyclopentanone, 2-(2-octenyl)- | Not found | 32.24 | C13H22O | 10 |
| 133 | iso-velleral | Isovelleral | 32.564 | C15H20O2 | 10 |
| 134 | iso-velleral | Isovelleral | 32.907 | C15H20O2 | 10 |
| 135 | allyl ionone 1 | Allyl Ionone | 33.71 | C16H24O | 10 |
| 136 | Cyclohexane, (1-Bromo-3,3-dimethyl-2-hydroxybutylidene)- | Not found | 34.152 | C12H21BrO | 6 |
| 137 | Androstan-17-one, 3-ethyl-3-hydroxy-, (5.alpha.)- | Androstan | 34.34 | C21H34O2 | 10 |
| 138 | Ethyl 5,8,11,14,17-eicosapentaenoate | Not found | 35.817 | C22H34O2 | 10 |
| 139 | 2-(4a,8-Dimethyl-1,2,3,4,4a,5,6,7-octahydro-naphthalen-2-yl)-prop-2-en-1-ol | Not found | 36.021 | C15H24O | 10 |
| 140 | 1,2-Benzenedicarboxylic acid, bis(2-ethylhexyl) ester | DEHP/ DNOP | 39.649 | C24H38O4 | 10 |
| 141 | Cholest-5-ene, 3-bromo-, (3.beta.) | Not found | 48.262 | C27H45Br | 10 |
